# Improvement of Emotional Empathy and Cluster B Personality Disorder Symptoms Associated With Decreased Cocaine Use Severity

**DOI:** 10.3389/fpsyt.2019.00213

**Published:** 2019-04-05

**Authors:** Matthias Vonmoos, Christoph Eisenegger, Oliver G. Bosch, Katrin H. Preller, Lea M. Hulka, Markus Baumgartner, Erich Seifritz, Boris B. Quednow

**Affiliations:** ^1^Experimental and Clinical Pharmacopsychology, Department of Psychiatry, Psychotherapy and Psychosomatics, Psychiatric Hospital of the University of Zurich, Zurich, Switzerland; ^2^Neuropsychopharmacology and Biopsychology Unit, Department of Basic Psychological Research and Research Methods, Faculty of Psychology, University of Vienna, Vienna, Austria; ^3^Department of Psychiatry, Psychotherapy and Psychosomatics, Psychiatric Hospital of the University of Zurich, Zurich, Switzerland; ^4^Center of Forensic Hairanalytics, Institute of Forensic Medicine, University of Zurich, Zurich, Switzerland; ^5^Neuroscience Center Zurich, University and ETH Zurich, Zurich, Switzerland

**Keywords:** cocaine, stimulants, social cognition, empathy, Theory-of-Mind, social decision-making, cognition, personality disorder

## Abstract

**Aims:** Chronic cocaine users display impaired social cognitive abilities, reduced prosocial behavior, and pronounced cluster B personality disorder (PD) symptoms all contributing to their social dysfunctions in daily life. These social dysfunctions have been proposed as a major factor for maintenance and relapse of stimulant use disorders in general. However, little is known about the reversibility of social cognitive deficits and socially problematic personality facets when stimulant use is reduced or ceased. Therefore, we examined the relation between changing intensity of cocaine use and the development of sociocognitive functioning and cluster B PD symptomatology over the course of 1 year.

**Methods:** Social cognition, social decision-making, and cluster B PD symptoms were assessed in 38 cocaine users (19 with increased and 19 with decreased use) and 48 stimulant-naive healthy controls at baseline and at 1-year follow-up. Cocaine use severity was objectively determined by quantitative 6-month hair analyses. The categorization of the two cocaine user groups was based on a combination of absolute (± 0.5 ng/mg) and relative (± 10%) changes in the cocaine hair concentration between baseline and the 1-year follow-up. Social cognition was assessed using the Multifaceted Empathy Test (MET) and the Movie for the Assessment of Social Cognition (MASC). A combined Distribution/Dictator Game was applied for assessing social decision-making. Cluster B PD symptoms were measured by a Structured Clinical Interview for DSM-IV Axis II Disorders (SCID-II) PD questionnaire according to *Diagnostic and Statistical Manual of Mental Disorders*, 4th edition (DSM-IV).

**Results:** Increased cocaine use was linked to worsened empathy, while decreased cocaine use went along with improved emotional empathy. Moreover, whereas decreased cocaine use was associated with reduced severity of self-reported cluster B PD symptoms, these symptoms remained largely stable in *increasers*. In contrast to a significant reduction of prosocial behavior at baseline in the combined cocaine user group, specifically *decreasers* were not statistically distinguishable from controls at the follow-up.

**Conclusions:** Sociocognitive deficits and cluster B PD symptoms of chronic cocaine users are adaptable over time as they covary with the increase or decrease in cocaine use. Hence, abstinence orientation and training of social cognition and interaction might improve social functioning, and should therefore be important therapeutic elements in cocaine addiction treatment.

## Introduction

Neurocognitive deficits such as impaired attention, memory, and executive functions related to chronic cocaine use are well documented (1–3) and a risk factor for poor treatment outcomes (4, 5). While some studies investigated the linkage between these neurocognitive deficits and cocaine abstinence (6), only one study yet investigated the longitudinal relationship between cognitive impairments and changing cocaine use (7). In sum, these studies indicate that basal cognitive deficits in cocaine users seem to be largely drug-induced, remain stable during the first weeks of abstinence but likely improve after some months (8).

While nonsocial cognitive functions have been studied well during the last two decades, the systematic assessment of sociocognitive functioning in cocaine users has only recently emerged. Per definition, the concept of social cognition comprises not only abilities enabling the dynamic interaction with our social environments and include emotional and mental perspective-taking functions such as emotion recognition, emotional empathy (EE), and *Theory-of-Mind*, but also interactive abilities such as social decision-making (SDM), moral behavior, and social network behavior (9, 10). As daily-life social functioning strongly depends on intact social cognition and as the deteriorative impact of sociocognitive impairments on development, progress, and prognosis on other psychiatric disorders such as schizophrenia is well known (11), a close relationship between sociocognitive functioning and the origin and course of stimulant use disorders has been proposed (12–15). Accordingly, we previously demonstrated smaller social networks (16), reduced EE (16), altered SDM (17), stronger detachment from social norms (14), and impaired emotion recognition from voices (18) in recreational and dependent cocaine user groups. Moreover, dependent cocaine users made more errors than controls in a video-based *Theory-of-Mind* task, with recreational cocaine users performing intermediate between the two groups (16). Finally, cocaine users show also blunted neuronal responses to implicit and explicit forms of social reward (19, 20). Notably, all these studies were implemented with a cross-sectional design, but no study has investigated the longitudinal development of sociocognitive functioning so far. Thus, it is unclear if sociocognitive impairments are predisposed or drug-induced and if they are reversible upon prolonged abstinence or reduction of drug use.

As social cognition is the sum of those processes that allow individuals to interact in interpersonal contexts (21), disturbed sociocognitive functioning leads to aberrant social behavior and, in excessive forms, to deviant personality characteristics and impaired interpersonal functioning (22, 23). Notably, cocaine-addicted individuals show an increased risk for concurrent cluster B personality disorders (PDs), mainly of the antisocial and borderline types (24, 25). A cluster B PD comorbidity is largely influential for cocaine addiction severity and treatment outcomes including pronounced executive function deficits (26), more intense cocaine intake, lower rates of treatment applications, and decreased probability of cocaine addiction remission (27, 28). Additionally, it was demonstrated that impulsivity and gambling decision-making, which are both closely related to cluster B PD pathologies (22, 29), covary with changes in the intensity of cocaine use over 1 year (30). Nonetheless, the longitudinal relation between cocaine use intensity and cluster B PD symptomatology has also not been investigated to date.

In sum, only little is known about the temporal dynamics between cocaine use intensity and sociocognitive functioning. Hence, in order to investigate whether the described sociocognitive impairments and comorbid cluster B PD symptomatology in chronic cocaine users are modulated by the increase or decrease in cocaine abuse, we performed a longitudinal study with an interval of 1 year. Thereby, we compared 48 psychostimulant-naive controls with 19 cocaine users with decreased use (*decreasers*) and 19 cocaine users with increased use (*increasers*) after a 1-year interval. To objectively assess the severity and change in cocaine use and to control for co-use of other drugs, we performed quantitative hair and urine toxicology analyses at baseline and follow-up. Considering our previous results from the present sample that changes in basal cognitive functions and impulsivity clearly covary with cocaine use intensity over time (7, 30), we hypothesized that escalating cocaine use is also associated with aggravation of sociocognitive impairments and more cluster B PD symptoms within 1 year. *Vice versa*, we also expected that reduced cocaine use is linked to a reduction of sociocognitive deficits and cluster B PD symptomatology. To test these hypotheses, we expect significant time × group interactions specifically between *decreasers* and *increasers*. Given that at baseline cocaine users displayed significant alterations in EE, social network size, prosocial behavior in money distribution games, *Theory-of-Mind*, and cluster B PD symptoms (14, 16, 17), the longitudinal analysis was focused solely on these parameters.

## Methods

### Participants

From a baseline sample of 234 participants (96 healthy stimulant-naive controls, 138 cocaine users) (3, 16, 17), 48 healthy stimulant-naive controls and 38 chronic cocaine users were included in the present longitudinal study. This subsample has been published twice previously but with different outcome measures (7, 30). From the baseline sample, 102 participants could not be measured at the follow-up because of unavailability (i.e., not responding to the invitation, loss of interest, lack of time, death), 27 participants had to be excluded from the final analyses as hair analyses revealed drug use not allowed by our exclusion criteria (e.g., polysubstance use, change in drug preferences), and 19 cocaine users did not meet our cocaine use criteria [see also the cocaine user group assignment below; for further recruitment and selection details, please see Ref. (7)].

At baseline, general exclusion criteria were clinically significant somatic diseases, neurological disorders, head injuries, family history of schizophrenia/obsessive-compulsive disorder/bipolar disorder, or any medication affecting the central nervous system. Additional exclusion criteria for controls were *Diagnostic and Statistical Manual of Mental Disorders*, 4th edition (DSM-IV) axis I psychiatric disorders (excluding nicotine dependence) and regular illegal drug use (>15 lifetime occasions, except for recreational cannabis use). Additional exclusion criteria for cocaine users were a history of heroin use, polysubstance use, or DSM-IV axis I psychiatric disorders (except for cocaine, nicotine, cannabis, and alcohol abuse/dependence, attention deficit hyperactivity disorder, and a previous episode of an affective disorder). At baseline, inclusion criteria for cocaine users were cocaine use of >0.5 g per month, cocaine as primary drug, and an abstinence duration of <6 months. Participants were asked to abstain from illegal substances for at least 72 h and from alcohol for 24 h before the test sessions. Compliance with these instructions was controlled by urine screenings (semiquantitative enzyme multiplied immunoassay method). The study was approved by the Cantonal Ethics Committee of Zurich. All participants provided written informed-consent statements and were compensated for their participation.

### Cocaine User Group Assignment

The categorization of the two cocaine user groups was based on changes of cocaine concentration in hair samples as determined by liquid chromatography–tandem mass spectrometry [for technical details, see Ref. (3)]. If possible, 6-cm hair samples were drawn covering the previous drug use of approximately 6 months. Cocaine users were categorized based on a combination of absolute (±0.5 ng/mg) and relative (>10% increase/decrease) changes in the hair concentration of cocaine_total_ between baseline and the 1-year follow-up (7, 30, 31). According to these criteria, cocaine users were divided into three equally sized groups: 19 cocaine *increasers* [mean ± SD: +30.4 ± 61.9 ng/mg (+297%), range: +0.5 to +268.5 ng/mg (+20% to +5,374%)], 19 cocaine *decreasers* [−10.6 ± 26.7 ng/mg (−72%), −116.9 to −0.6 ng/mg (−100% to −12%)]), and 19 users with a relatively low and stable cocaine use pattern who did not meet both criteria [−0.1 ± 0.5 ng/mg (−2%), −1.9 to +0.5 ng/mg (−100% to +720%)], and, thus, were not further analyzed in this study [for further details, see Ref. (7)].

### Procedure

At baseline, self-reported drug use was assessed with a structured and standardized Interview for Psychotropic Drug Consumption (32), attention deficit hyperactivity disorder (ADHD) symptoms were assessed with the ADHD Self-Rating Scale (ADHD-SR) (33), and the Structured Clinical Interview for DSM-IV axis I disorders (SCID-I) (34) was carried out by trained psychologists.

The test battery was assessed at baseline and follow-up and included the Multifaceted Empathy Test (MET) (35) assessing EE, the Movie for the Assessment of Social Cognition (MASC) (36) for the measurement of *Theory-of-Mind*, a Distribution/Dictator Game (37, 38) for the determination of prosocial behavior, the Social Network Questionnaire (SNQ) (39) measuring the social network size, and the SCID-II questionnaire (40) in order to ascertain cluster B PD symptoms. More detailed test descriptions published already in our previous work (16, 17) are given in **Methods S1**.

### Statistical Analysis

Effect sizes and power analyses were calculated with G*Power 3.1 (41). As our previous analyses showed an effect size of _p_η^2^ = 0.12 (Cohen’s f = 0.37) and a power of 99% for the significant interaction in the domain of working memory between *decreasers* and *increasers* (two groups, p < .05, two measurements) for the present sample (7), we assumed a more conservative effect size of _p_η^2^ = 0.06 (f = 0.25) and calculated a still acceptable power of 86% for the detection of significant interactions in sociocognitive functions in the present sample.

In order to reduce data quantity [see also Ref. (17)], we computed an SDM composite score that was derived by averaging z-transformed payoffs for the other player in the Distribution and Dictator Game (payoffs B) according to the means and standard deviations of the control group. Because of a strong correlation of the explicit and implicit EE scores from the MET in the total sample (r = 0.86, p < .001), we further integrated both parameters by adding them up into a single MET EE score. The SCID-II Cluster B symptom score was calculated by summing up the dimensional values from histrionic, narcissistic, borderline, and antisocial PD.

Group differences in demographic data and drug use patterns were analyzed by means of Pearson’s chi-squared tests, analyses of variance (ANOVA), or independent Student’s t-tests. For the longitudinal analysis and in order to investigate group differences over all groups, we performed a multiple linear regression (forced entry) with the test score change values (Δ = t2 − t1) as dependent variables and four preselected independent variables: age, sex, ADHS-SR score, and dummy-coded (zero/one) group contrasts. The two demographic variables were included because previous findings suggest a linkage between advancing age and fairness in stimulant users (17) and due to known gender effects in social cognition/functioning (42, 43). Moreover, because ADHD has previously been linked to cognitive and sociocognitive performance in cocaine users (3, 16, 44), this variable was further included as a predictor into the regression model. To compare the groups, cocaine *increasers* acted as the reference group. To further analyze test score changes within the single groups (value t2 vs. value t1), we applied dependent Student’s t-tests (t_dep_). To compare the effect of changing cocaine use, we applied independent Student’s t-tests (t_ind_) between controls and a combined cocaine user sample (CCU = *increasers* + *decreasers*) at baseline as well as between controls, cocaine *increasers*, and cocaine *decreasers* at the follow-up. Notably, at baseline, cocaine *increasers* and *decreasers* showed comparable baseline values in all reported test parameters (MET, MASC, SDM, SNQ, SCID-II Cluster B) differing only with very small effect sizes (t_ind_(32–35) = 0.05–0.34, p = .99–.74, d = 0.00–0.11). In the test parameter analysis, frequency data were analyzed by the Fisher–Freeman–Halton Exact Test (FET) (45). To test for test–retest effects, we applied the Pearson product-moment correlation analyses. The confirmatory statistical comparisons were carried out on a significance level of p < .05 (two-tailed).

## Results

*Demographic characteristics and drug use:* As shown before (7, 30), the three experimental groups did not significantly differ regarding age, sex distribution, verbal IQ, years of education, length of study interval (**Table 1**), and socioeconomic status (**Table S1**). Still, cocaine-using groups showed significantly higher BDI and ADHD-SR sum scores than controls at baseline (7, 30). Whereas at baseline both cocaine user groups showed comparable cocaine use severity, the cocaine_total_ hair concentrations for *increasers* (∼3-fold increase) and *decreasers* (reduction by the factor 3.5) were significantly different at follow-up. Moreover, hair data revealed a clear preference for cocaine use compared to other illegal drugs. Finally, in both user groups, 8 of 19 participants sought psychiatric or psychological treatment during the study interval. The other cocaine users did not report any related treatment between baseline and follow-up.

**Table 1:** Demographic data and pattern of cocaine use.

	Baseline (t1)						1-year follow-up (t2)^m^					
	Controls (n = 48)	Cocaine Increaser (n = 19)	Cocaine Decreaser (n = 19)	F/χ²/T	df, df_err_	p	Controls (n = 48)	Cocaine Increaser (n = 19)	Cocaine Decreaser (n = 19)	F/χ²/T	df, df_err_	p
Age, years	30.3 (8.9)	31.5 (9.4)	31.4 (8.3)	.20^a^	2,83	.82						
Sex (f/m)	16/32	3/16	5/14	2.11^b^	2	.35						
Verbal IQ (MWT-B)^d^	107.6 (10.0)	102.9 (9.7)	103.8 (7.1)	2.20^a^	2,83	.12						
Education, years	10.8 (1.8)	10.4 (1.8)	10.0 (1.5)	1.30^a^	2,83	.28						
ADHD-SR score (0-22)	7.7 (5.2)	13.5 (9.4)**	14.1 (6.8)**	8.83^a^	2,83	** <.001**						
ADHD DSM IV (y/n)^e^	0/48	4/15	3/16	7.02^b^	2	**.03**						
Weeks between t1 and t2	58.2 (10.1)	59.3 (12.1)	61.9 (14.5)	.69^a^	2,83	.50						
BDI score (0–63)	3.5 (3.3)	7.3 (8.0)*	8.7 (6.5)**	7.53^a^	2,83	** <.001**						
BDI depression (y/n)^g^	0/48	1/18	1/18	2.59^b^	2	.27						
**Cocaine**												
Times per week^h^	–	1.6 (1.8)	1.0 (1.3)	1.17^c^	36	.25	–	1.1 (0.8)	0.3 (0.3)	3.85^c^	36	**<.001**
Grams per week^h^	–	2.0 (2.5)	1.7 (2.3)	.41^c^	36	.68	–	1.6 (2.5)	0.4 (0.4)	2.18^c^	36	**.04**
Years of use	–	7.0 (5.5)	8.2 (5.4)	.68^c^	36	.50	–	8.9 (5.4)	9.7 (5.2)	.45^c^	36	.65
Age of cocaine onset	–	24.5 (8.1)	23.1 (5.2)	.61^c^	36	.54						
Max. dose (g/day)^k^	–	4.7 (4.4)	5.9 (6.4)	.71^c^	36	.48	–	3.7 (2.5)	3.1 (2.8)	.63^c^	36	.53
Cumulative dose (g)^k^	–	1182 (1635)	3698 (8585)	1.25^c^	36	.22	–	91 (119)	49 (89)	1.25^c^	36	.22
Last consumption (days)	–	18.5 (25.1)	16.8 (14.6)	.29^c^	36	.77	–	7.0 (6.3)	81.4 (145.1)	2.23^c^	36	**.03**
Cocaine craving (0–70)^i^	–	19.8 (9.5)	17.7 (7.2)	.79^c^	36	.44	–	20.5 (10.8)	15.8 (6.2)	1.66^c^	36	.11
Hair analysis (ng/mg)^l^												
Cocaine_total_	–	10.3 (29.2)	14.9 (32.2)	.46^c^	36	.65	–	40.7 (76.1)	4.2 (8.2)	2.08^c^	36	**.05**
Cocaine	–	8.2 (23.3)	11.4 (23.9)	.42^c^	36	.68	–	31.7 (56.5)	3.1 (5.9)	2.19^c^	36	**.03**
Benzoylecgonine	–	1.9 (5.5)	3.1 (7.6)	.58^c^	36	.56	–	8.3 (19.6)	1.0 (2.2)	1.62^c^	36	.11
Cocaethylene	–	1.0 (2.8)	0.9 (2.8)	.11^c^	36	.91	–	1.2 (2.1)	0.3 (1.0)	1.56^c^	36	.13
Norcocaine_t_	–	0.2 (0.5)	0.4 (0.8)	.83^c^	36	.41	–	0.6 (1.4)	0.1 (0.1)	1.71^c^	36	.10
Urine toxicology (n/p)^k^	48/0	14/5	16/3	.63^b^	1	.43	48/0	7/12	18/1	14.15^b^	1	**<.001**
**Alcohol** ^n^												
Grams per week^h^	119.9 (136.8)	169.4 (129.2)	155.3 (146.4)	1.07^a^	2,83	.35	104.3 (88.6)	259.7 (244.5)***	127.4 (141.4)°	7.71^a^	2,83	**<.001**
Years of use	13.3 (8.8)	13.7 (7.6)	12.0 (7.3)	.23^a^	2,83	.79	14.0 (8.7)	14.8 (7.5)	12.6 (7.9)	.34^a^	2,83	.71
**Nicotine** ^n^												
Smoking (y/n)^f^	37/11	14/5	14/5	.13^b^	2	.94	40/8	15/4	13/6	1.83^b^	2	.40
Cigarettes per day^h^	8.7 (8.7)	12.8 (11.2)	9.5 (8.2)	1.38^a^	2,83	.26	8.2 (8.7)	13.4 (12.0)	8.2 (7.8)	2.31^a^	2,83	.11
Years of use	9.3 (8.3)	10.4 (8.9)	12.7 (10.3)	.95^a^	2,83	.39	10.5 (8.8)	12.5 (8.6)	12.6 (9.9)	.56^a^	2,83	.57
**Cannabis** ^n^												
Grams per week^h^	0.6 (1.6)	3.3 (8.9)	1.2 (2.3)	2.38^a^	2,83	.10	0.5 (1.6)	2.1 (4.6)	1.1 (2.7)	2.28^a^	2,83	.11
Years of use	4.5 (4.9)	9.5 (8.5)*	10.1 (9.7)*	5.92^a^	2,83	**.004**	4.6 (5.9)	10.5 (9.8)*	8.6 (9.7)	4.64^a^	2,83	**.01**
Cumulative dose (grams)	980 (3985)	3199 (5899)	2606 (6359)	1.61^a^	2,83	.21	53.4 (180)	217.8 (526.5)	84.7 (189.6)	2.15^a^	2,83	.12
Last consumption (days)^j^	39.3 (1.6);n = 22	10.0 (0.4);n = 14	25.4 (1.1);n = 12	2.19^a^	2,45	.12	36.5 (1.5);n = 22	9.7 (0.4);n = 13	50.8 (2.1);n = 10	1.20^a^	2,42	.31
Urine toxicology (n/p)^k^	42/6	15/4	14/5	2.03^b^	2	.36	42/6	7/12	15/4	18.61^b^	2	**<.001**
**Amphetamine** ^n^												
Grams per week^h^	0.0 (0.1)	0.1 (0.1)**	0.0 (0.1)	5.18^a^	2,83	**.008**	0.0 (0.0)	0.1 (0.2)**	0.0 (0.1)	5.89^a^	2,83	**.004**
Years of use	0.0 (0.0)	3.3 (4.0)***	1.3 (3.1)°	13.73^a^	2,83	** <.001**	0.1 (0.5)	3.2 (4.9)**	2.7 (5.5)*	7.46^a^	2,83	**.001**
Cumulative dose (grams)	0.0 (0.1)	56.0 (177.6)*	16.2 (35.9)	2.99^a^	2,83	.06	0.0 (0.1)	4.4 (8.9)**	1.4 (3.5)	6.47^a^	2,83	**.002**
Last consumption (days)^j^	121.6 (5.1);n = 1	73.6 (3.1);n = 10	90.9 (3.8);n = 3	.29^a^	2,11	.75	17.5 (0.7);n = 1	35.7 (1.5);n = 8	99.8 (4.2);n = 4	1.48^a^	2,10	.27
Hair analysis (ng/mg)	0.0 (0.0)	0.1 (0.2)*	0.0 (0.0)	4.35^a^	2,83	**.02**	0.0 (0.0)	0.1 (0.2)	0.1 (0.2)	2.89^a^	2,83	.06
**MDMA** ^n^												
Tablets per week^h^	0.0 (0.0)	0.0 (0.1)***	0.0 (0.0)°	7.42^a^	2,83	**.001**	0.0 (0.0)	0.4 (0.9)**	0.0 (0.0)°	5.54^a^	2,83	**.006**
Years of use	0.3 (1.0)	3.5 (4.5)***	2.4 (4.6)*	8.42^a^	2,83	**<.001**	0.2 (1.4)	3.8 (5.5)**	3.2 (5.6)*	7.78^a^	2,83	**<.001**
Cumulative dose (tablets)	1.3 (4.0)	108.8 (249.7)**	18.7 (46.2)	5.71^a^	2,83	**.005**	0.2 (0.8)	17.0 (49.3)*	2.8 (5.2)	3.67^a^	2,83	**.03**
Last consumption (days)^j^	5.0 (0.2);n = 1	89.9 (3.7);n = 7	40.2 (1.7);n = 4	1.63^a^	2,9	.25	91.2 (3.8);n = 3	41.6 (1.7);n = 6	47.8 (2.0);n = 5	1.11^a^	2,11	.36
Hair analysis (ng/mg)	0.0 (0.0)	0.3 (0.7)	0.4 (1.5)	2.23^a^	2,83	.11	0.0 (0.0)	0.5 (0.8)***	0.1 (0.3)	7.87^a^	2,83	**<.001**
**GHB** ^n^												
Cumulative dose (pipettes)	0.0 (0.0)	0.5 (0.7)	0.5 (1.7)	3.36^a^	2,83	**.04**	0.0 (0.0)	0.0 (0.0)	0.0 (0.0)	–	–	–
**Hallucinogens** ^n^												
Cumulative dose (times)	0.9 (2.2)	27.9 (72.8)*	9.9 (22.9)	3.92^a^	2,83	**.02**	0.0 (0.0)	1.1 (1.6)***	0.6 (1.5)	8.57^a^	2,83	**<.001**
**Methlyphenidate** ^n^												
Cumulative dose (tablets)	0.0 (0.0)	20.2 (60.4)*	0.5 (2.3)	3.76^a^	2,83	**.03**	0.0 (0.1)	67.7 (239.5)	0.3 (0.6)	2.72^a^	2,83	.07
Hair analysis (ng/mg)	0.0 (0.0)	0.0 (0.1)	0.0 (0.0)	1.80^a^	2,83	.17	0.0 (0.0)	0.1 (0.2)*	0.0 (0.0)	3.62^a^	2,83	**.03**

Means and standard deviations. Significant p values are shown in bold.

a ANOVA (all groups, with significant Sidak post hoc test vs. control group: *p < .05; **p < .01; ***p < .001; vs. cocaine increaser: p < .05).

b *χ*²-test (all groups/cocaine users only) for frequency data.

c Independent t-test (cocaine users only).

d Verbal IQ was assessed by the Mehrfachwahl Wortschatz Intelligenztest (46).

e ADHD-SR, ADHD self-rating scale (cutoff DSM-IV criteria) (33).

f Smoking habits were assessed by the Fagerstroem Test of Nicotine Dependence (47).

g BDI, Beck Depression Inventory (cutoff ≥ 18) (48).

h Average use during the last 6 months.

i Craving for cocaine was assessed by the Brief-CCQ (49).

j Last consumption is averaged only for persons who used the drug in the last 6 months.

k Urine toxicology (neg/pos) are based on the cutoff value for cocaine = 150 ng/ml and for tetrahydrocannabinol 50 ng/ml (50). The *χ*²-test for cocaine includes only cocaine users; the *χ*²-test for cannabis includes controls and cocaine users.

l Hair samples were voluntary and data are missing for three controls.

m Parameters at follow-up refer to the 1-year period between t1 and t2.

n At baseline, average use during the last 6 months. Use frequency, duration of use, and cumulative doses are averaged within the total group.

**Figure 1:**
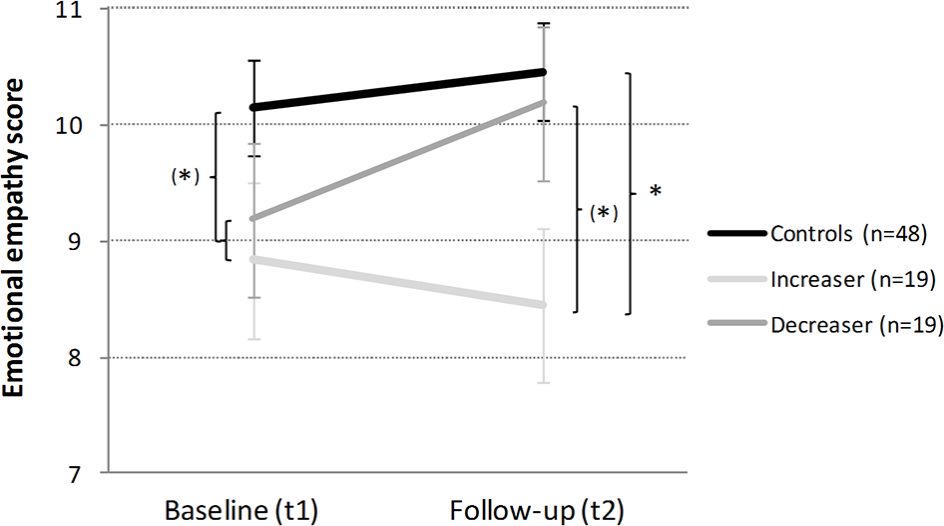
Development of emotional empathy in cocaine *increasers*, *decreasers*, and stimulant-naive controls within 1 year. Mean emotional empathy sum scores and standard errors. At baseline, controls vs combined cocaine user (CCU) (= Ø of increaser and decreaser). Independent Student’s t-tests are shown if p < .10. ^(^*^)^p < .10; *p < .05.

*Emotional empathy*: The introduced predictors explained a significant amount of variance of the EE change scores in the multiple regression analysis [F(5,80) = 2.68, p < .05, R^2^ = .14; **Table 2**]. The strongest predictors were sex (β = −0.25, p < .05) and the group contrast cocaine *increasers* vs. *decreasers* (β = −0.25, p < .05; **Figure 1**). *Post hoc* analyses showed that controls and the combined cocaine user (CCU) group showed a nonsignificant difference in EE with a small to moderate effect size at baseline [t_ind_(84) = 1.78, p = .08, d = 0.39]. Whereas in the 1-year interval, *increasers* slightly reduced their already hampered EE [t_dep_(18) = 1.19, p = .25 d = 0.27], and cocaine *decreasers* moderately improved their ability to respond empathically [t_dep_(18) = 1.80, p = .09, d = 0.41]. Notably, controls remained largely stable in EE [t_dep_(47) = 0.98, p = .33, d = 0.14]. Accordingly, at follow-up, controls differed significantly from the *increaser* group [t_ind_(25) = 2.14, p < .05, d = 0.64], whereas the difference between controls and *decreasers* was strongly reduced [t_ind_(65) = 0.40, p = .69, d = 0.11]. Finally, at follow-up, *increasers* and *decreasers* displayed a nonsignificant group difference of moderate effect size [t_ind_(36) = 1.69, p = .10, d = 0.56].

**Table 2:** Multiple regression analyses.

	Emotional empathy Δ MET Emotional empathy			Theory-of-Mind Δ MASC total errors			Social interaction Δ SDM composite score			Social network Δ SNQ network size			Personality disorder Δ SCID-II Cluster B		
	B	SE	β	B	SE	β	B	SE	β	B	SE	β	B	SE	β
Constant	1.55	1.14		−1.85	2.02		−0.26	0.45		−0.73	2.72		−1.01	2.80	
Age	0.04	0.03	0.14	0.06	0.05	0.14	−0.01	0.01	−0.08	−0.04	0.06	−0.08	0.02	0.06	0.04
Sex	−1.16	0.50	−0.25*	−0.99	0.86	−0.12	0.09	0.20	0.05	−0.56	1.20	−0.05	−0.71	1.23	−0.06
ADHD-SR score	−0.05	0.03	−0.16	−0.07	0.06	−0.14	0.00	0.01	−0.01	0.04	0.08	0.05	−0.24	0.08	−0.32**
Controls vs. cocaine increaser	−0.26	0.59	−0.05	1.71	1.03	0.20	0.03	0.24	0.01	0.35	1.43	0.03	4.00	1.49	0.31**
Cocaine decreaser vs. cocaine increaser	−1.30	0.66	−0.25*	−0.51	1.17	−0.06	−0.31	0.27	−0.16	0.54	1.63	0.05	4.43	1.69	0.34**
R^2^		0.14			0.10			0.03			0.01			0.17	
F		2.68*			1.67			0.57			0.22			3.25**	

Multiple linear regression with the independent variables age, sex, ADHS-SR score, and dummy coded (zero, one) group variables. To compare the groups, cocaine increaser acted as the reference group. Dependent variables are change values (Δ = value at t2 − value at t1).

B, unstandardized regression coefficient; SE, unstandardized standard error; *β*, standardized beta.

^(^*^)^p < .10; *p < .05; **p < .01; ***p < .001.

**Figure 2:**
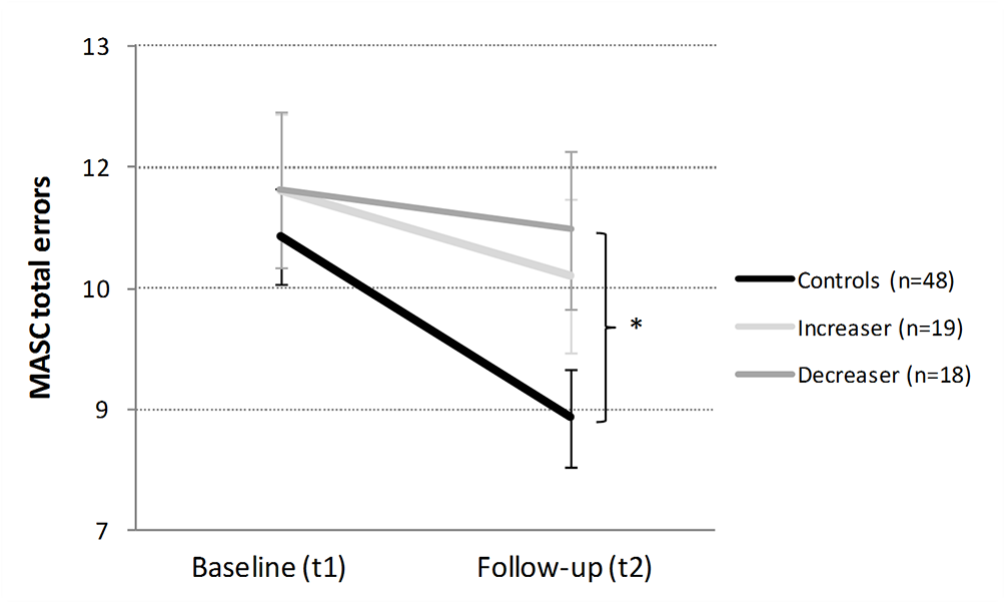
Development of the *Theory-of-Mind* in cocaine *increasers*, *decreasers*, and stimulant-naive controls within 1 year. Mean Movie for the Assessment of Social Cognition (MASC) total errors and standard errors. At baseline, controls vs CCU (= Ø of increaser and decreaser). Independent Student’s t-tests are shown if p < .10. *p < .05.

*Theory-of-Mind*: The applied multiple regression model could not predict the MASC total error change scores (**Table 2**). At the phenomenological level, both cocaine user groups displayed small test–retest improvements [*increasers*: t_dep_(18) = 1.12, p = .28, d = 0.26; *decreasers*: t_dep_(17) = 0.60, p = .56, d = 0.14], while the control group showed pronounced improvements [t_dep_(47) = 4.68, p < .001, d = 0.68; **Figure 2**).

**Figure 3:**
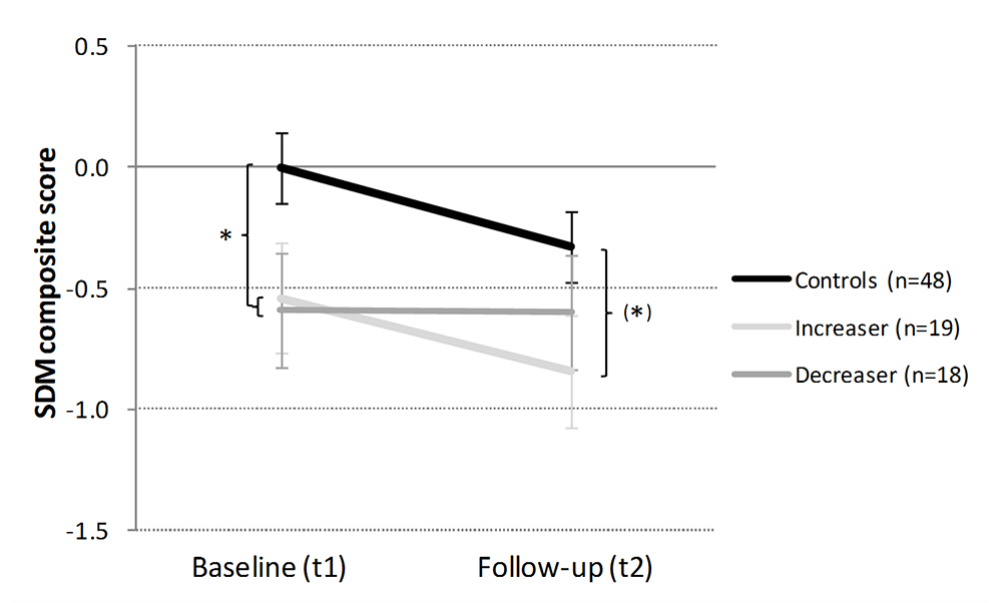
Development of social interaction in cocaine *increasers*, *decreasers*, and stimulant-naive controls within 1 year. Mean social decision-making (SDM) composite z-scores and standard errors. At baseline, controls vs CCU (= Ø of increaser and decreaser). Independent Student’s t-tests are shown if p < .10. ^(^*^)^p < .10; *p < .05.

*Social interaction*: The multiple regression model was also not able to predict the SDM composite change score (**Table 2**). From the phenomenological perspective, controls and *increasers* acted less prosocial (giving less money to the opponent), while *decreasers* remained stable but, with that, came closer to the controls (**Figure 3**). Exploratory *post hoc* analyses confirmed that controls and CCU significantly differed at baseline [t_ind_(65) = 2.51, p < .05, d = 0.56]. At follow-up, controls and *increasers* still display a moderate group difference [t_ind_(65) = 1.92, p = .06, d = 0.50], whereas the group difference between controls and *decreasers *was reduced to a small effect size [t_ind_(64) = 0.98, p = .33, d = 0.26]. In addition, we analyzed behavioral changes between baseline and follow-up (more prosocial decisions, more self-serving decisions, similar decision) only in cocaine users and found that about two-thirds of the *increasers* (58% = 11/19) but only one-third of the *decreasers* (33% = 6/18) showed more self-serving decisions at follow-up (p = .40; FET; **Figure S1**).

**Figure 4:**
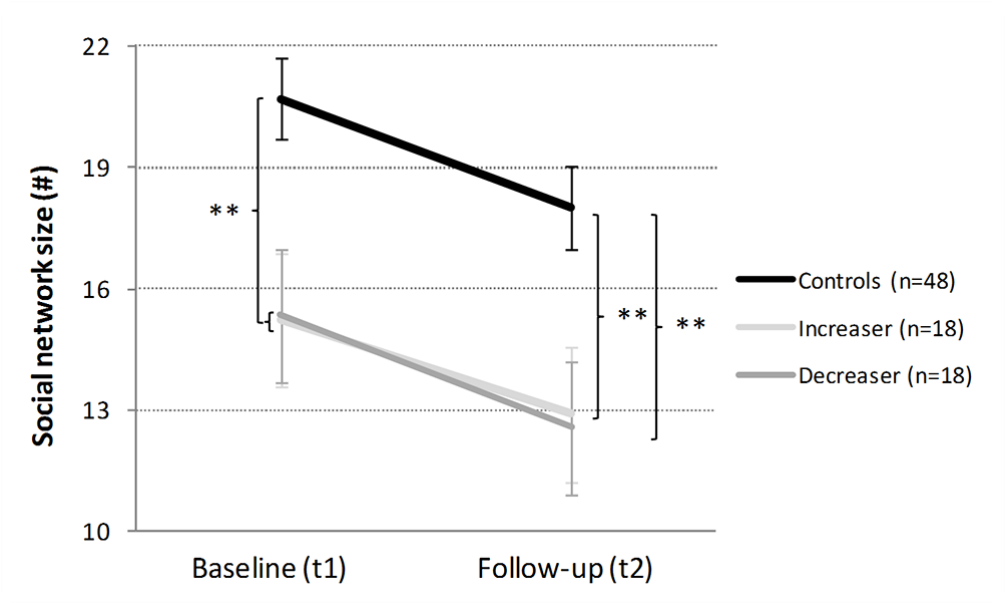
Development of social network size in cocaine *increasers*, *decreasers*, and stimulant-naive controls within 1 year. Mean total network size and standard errors. At baseline, controls vs CCU (= Ø of increaser and decreaser). Independent Student’s t-tests are shown if p < .10. **p < .01.

*Social network size*: Regarding the SNQ total network size, the multiple regression model could again not substantially predict the change scores (**Table 2**, **Figure 4**). Interestingly, during the 1-year interval, all three groups reported a substantial and moderate social network reduction of about 2.5 contacts [controls: t_dep_(47) = 3.75, p < .001, d = 0.54; *increasers*: t_dep_(17) = 1.94, p = .70, d = 0.46; *decreasers*: t_dep_(17) = 3.09, p < .01, d = 0.73].

**Figure 5:**
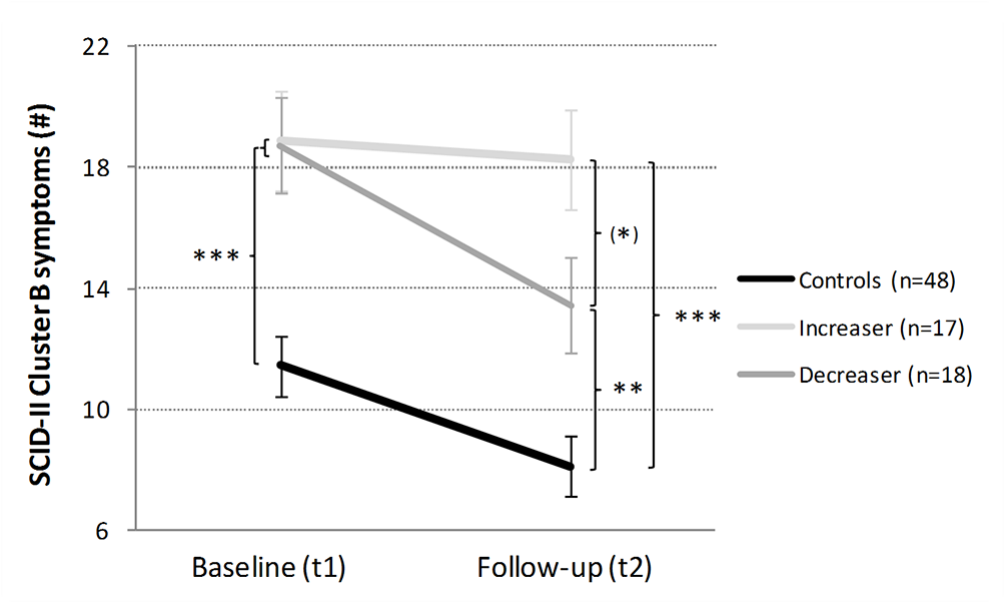
Development of Structured Clinical Interview for DSM-IV Axis II Disorders (SCID-II) Cluster B in cocaine *increasers*, *decreasers*, and stimulant-naive controls within 1 year. Mean SCID-II Cluster B symptoms and standard errors. At baseline, controls vs CCU (= Ø of increaser and decreaser). Independent Student’s t-tests are shown if p < .10. ^(^*^)^p < .10; **p < .01; ***p < .001.

*Cluster B PD*: The regression model significantly explained the variance in cluster B PD symptom change [F(5,77) = 3.25, p < .01, R^2^ = .17; **Table 2**]. This change score was best predicted by the ADHS-SR score (β = −0.32, p < .01) and the group contrasts cocaine *increasers* vs. *decreasers* (β = 0.34, p < .01) and cocaine *increasers* vs. controls (β = 0.31, p < .01). Importantly, the CCU group showed at baseline significantly more cluster B PD symptoms than the controls [t_ind_(81) = 4.40, p < .001, d = 0.96; **Figure 5**]. Whereas controls [t_dep_(47) = 4.91, p < .001, d = 0.71] and* decreasers* [t_dep_(17) = 3.55, p < .01, d = 0.84] had significantly lower symptom scores after the 1-year interval period, the amount of symptoms for the *increaser* group remained largely stable [t_dep_(16) = 0.52, p = .61, d = 0.13]. Accordingly, at follow-up, controls differed strongly from the* increasers* [t_ind_(19) = 4.70, p < .001, d = 1.58] and from the *decreasers* [t_ind_(22) = 3.11, p < .01, d = 0.96]. Interestingly, already after 1 year of different cocaine use, *increasers* and *decreasers* displayed a moderate to strong group difference in cluster B PD symptoms at follow-up [t_ind_(33) = 1.85, p = .07, d = 0.63].****Of note, approximately three quarters of the *decreasers* (13/18) displayed lower cluster B PD scores, while more than half of the cocaine *increasers* (9/17) showed even more symptoms at follow-up (p < .05; FET; **Figure S2)**.

Remarkably, the interaction effect on cluster B PD symptoms was mainly driven by changes in the narcissistic and borderline subscores and less by the histrionic and surprisingly also not by the antisocial subscore (see **Figure S3a–d**). Both the narcissistic and the borderline subscores revealed significant regression models (**Table S2**), but only the borderline subscore was significantly predicted by the group contrast cocaine *increasers* vs. *decreasers* (β = 0.40, p < .01). Compared to baseline, less symptoms occurred in controls [t_dep_(47) = 4.99, p < .001, d = 0.72] and *decreasers* [t_dep_(17) = 3.16, p < .01, d = 0.75] at follow-up, while symptoms remained stable in *increasers* [t_dep_(16) = 0.18, p = .86, d = 0.04], resulting in a strong group effect between *increasers* and *decreasers* [t_ind_(33) = 2.57, p < .05, d = 0.87] at follow-up.

*Change in alcohol use*: As not only cocaine but also alcohol intake was increased in *increasers *(see **Table 1**), the change in alcohol consumption was considered in additional multiple regression models. However, alcohol change was not significant in any of the main regression models (p-values ranged from .222 to .659) shown in **Table 2**, while the interaction effects and also the explained variances remained stable, indicating that changes in alcohol consumption have not impacted our main results.

*Test–retest reliability*: In the total sample, all dependent variables displayed acceptable to good test–retest reliabilities (**Table 3**). Interestingly, in the SDM paradigm, controls and CCU differed significantly in their test–retest reliability (z = −3.25; p < .001): While in controls the SDM score showed hardly acceptable reliability (r = 0.48; p < .001), it was good in the cocaine users (r = 0.85; p < .001).

**Table 3:** One-year test–retest reliability between baseline and 1-year follow-up in controls and cocaine users.

	Controls (n = 48)	Combined cocaine users (n = 38)	Total sample (n = 86)
MET Emotional empathy score	0.66***	0.79***	0.74***
MASC total errors	0.67***	0.60***	0.63***
SDM composite score	0.48***	0.85***	0.70***
SNQ network size	0.75***	0.81***	0.80***
SCID-II Cluster B symptoms	0.68***	0.74***	0.77***

Pearson’s product-moment correlation. Significance level: ***p < .001.

## Discussion

The present longitudinal study investigated the change of social cognition, social interaction, and socially relevant cluster B PD symptoms in healthy controls and relatively pure and non-help-seeking chronic cocaine users who clearly increased or decreased their cocaine consumption during a 1-year study interval. The most striking findings were that i) improved EE correlated with decreased cocaine consumption, whereas increased cocaine use severity was linked to less EE; and ii) cluster B PD symptom burden was lowered in *decreasers,* whereas *increasers* showed stable severity in these symptoms. Additionally, during the study interval, we found an approximation between controls and *decreasers* regarding their prosocial behavior, while the large gap between *increasers* and controls remained. Moreover, neither the *Theory-of-Mind* Task (MASC) nor the social network size showed interactions with changing cocaine use, indicating that mental perspective-taking (sometimes also interpreted as *cognitive empathy*) and the number of social contacts in the last months were not affected by changing drug use during the study interval.

Importantly, the present analysis of smaller (longitudinal) subgroups from our larger cross-sectional ZuCo^2^St sample published previously (14, 16, 17) still showed significantly reduced prosocial behavior, a smaller social network, and strongly elevated cluster B personality symptoms in the total group of cocaine users at baseline, indicating that these indicators of social functioning were robustly altered in this population. The EE score of the MET showed only a statistical trend between cocaine users and controls at baseline, but the present effect size (d = 0.39) was in the range of the previously reported effect sizes of the larger cross-sectional sample of recreational and dependent cocaine users (d = 0.39–0.64), suggesting rather a deficiency of power than a lack of reliability. This assumption is further supported by the fact that the MET EE score showed good test–retest reliability scores. Moreover, the MASC did not show any baseline group differences in the present subsample of cocaine users underscoring our previous conclusion that mainly very severe cocaine users with a putative ADHD comorbidity show disturbances in this task (16, 44).

While sociocognitive functions represent basic abilities in perspective-taking and interaction, more conventional psychopathology is aiming at the identification and quantification of symptoms in psychiatric disorders (51). As such, the research on the relationship between PDs and cocaine use is of special interest, as the differentiation between predispositions vs. drug-induced effects merges with the question if these pathologies are reversible or not. In our longitudinal investigation, we found that *decreasers* of cocaine consumption also significantly improved in cluster B PD symptoms during 1 year, whereas the increasers showed a stable PD symptom burden. This is insofar interesting as in both user groups 8 of 19 participants sought psychiatric treatment in the interval, but only *decreasers* improved in some social functions and socially relevant PD symptoms.

In general, PDs are defined as typical constellations of impaired subjective and behavioral traits that result in suffering of the affected individual and/or society (52). These personality traits are regarded as relatively stable across time and consistent across situations (diagnostically mandatory) (53–56). Moreover, cluster B PDs show a higher stability over 12 to 18 years than the other clusters (57). However, studies also found considerable variability of PD symptoms across individuals over time (58, 59), questioning the trait-like character of the disorder. An early study showed changes in PD symptoms related to treatment in substance-dependent patients (60). Interestingly, clusters A and C profited most, while cluster B changes were only observed in patients with borderline PD. In patients with cocaine addiction, cluster B PDs are the most frequent and these patients have the most severe courses of illness including worst treatment outcomes (24, 26–28, 61, 62). Therefore, cluster B PD symptoms are likely personality features that increase the risk for cocaine use and the development of an addiction. However, as seen in the present study, cluster B PD symptom load is nevertheless variable and reduction of consumption leads to a substantial improvement in these symptoms. Consequently, a reduction of cluster B PD symptom burden again increases likelihood of successful treatment, offering the patient an opportunity to leave the vicious circle of addiction.

The suggested consumption-dependent variability of social behavior as well as cluster B symptoms are well in line with our previous analyses from this sample that not only basal cognitive functions such as working memory but also self-reported impulsivity improve with a strong reduction of cocaine use, while they are worsened with increased cocaine consumption (7, 30). The present data and the previous analyses from this sample are also in accordance with our recent results from an independent longitudinal investigation showing that decreased cortical thickness (CT) of several regions within the prefrontal cortex of cocaine users can improve after a strong reduction of cocaine use, while sustained use went along with a further decrease in prefrontal CT during the study interval (63). Importantly, the cortical changes were correlated with cognitive changes, i.e., improved CT as associated with enhanced sustained attention (63). Thus, the overall pattern of change shown by longitudinal data supports our assumption that sociocognitive impairments of cocaine users are at least in part drug-induced and that neuroplastic changes in brain regions and neurotransmitter systems involved in social cognition, social interaction, and social reward processing contribute to a further decrease in social contact and social support leading to an increase in social isolation, aggression, and depressive symptoms. This ends in a further reduction of social reward resources, ongoing social withdrawal, and the establishment of cocaine as the main source of reward resulting in the maintenance of stimulant use and recurrent relapses (15).

While EE is more a perceptive social cognition ability, social decision-making (here assessed with a combined Distribution/Dictator Game) is a form of socially interactive behavior. In our previous cross-sectional analysis sample, cocaine users cared more about efficiency than about fairness compared to healthy controls at baseline (17). This was previously interpreted as predisposition of stimulant use (15), as such fairness preferences and severity of cocaine use were not correlated (17). Intriguingly, utilitarian and opportunistic attitudes assessed with the Machiavellianism Questionnaire (MACH-IV) were also increased in cocaine users compared to controls and were shown to be stable and independent of changing cocaine use (14). However, our data indicated a shift toward improved prosocial behavior in cocaine *decreasers* indicating space for enhancement potential by treatment. Conclusively, SDM deficits in cocaine users likely have both a trait and a state component, and it might be worse to specifically target the state component in therapy in order to improve the treatment outcome.

### Limitations

When interpreting the present results, some limitations of our study have to be considered: i) The total sample size is moderate for a longitudinal analysis. Moreover, the test–retest reliabilities of the applied social cognition tasks and questionnaire have a broad range (in controls: r = 0.48–0.75; in cocaine users: r = 0.60–0.85; in the total sample: r = 0.63–0.80). As a consequence of both, the shown interaction effects are not very strong (in terms of p-values). However, to our knowledge, these are the only existing longitudinal samples of chronic cocaine users with objectively verified increasing and decreasing cocaine use (by hair testing). Moreover, the included individuals were preferably pure cocaine users with little axis-I psychiatric comorbidities. We therefore think that the carefully selected and homogeneous sample has nonetheless sufficient explanatory power. ii) In the context of our hypotheses, we attribute the changes in behavior to the changes in cocaine consumption. However, we cannot rule out if other changes in the lives of our cocaine users (e.g., positive or negative changes in their social environment) not assessed by our test battery may have impacted both drug use and social functioning. Future studies should therefore assess more information on the social life of cocaine users beyond simple parameters such as social network size (e.g., social media use).

### Conclusions

The aim of this longitudinal study was to investigate whether cocaine use is associated with permanent or reversible alterations of social cognition and interaction as well as cluster B PD symptoms. We found that specific social dysfunctions and PD symptoms are variable over time as they seem to depend on variations in cocaine use. Thus, strong reduction of cocaine use within only 1 year seems to positively affect social dysfunctions that are assumed to be crucial factor in the maintenance of stimulant addiction (15, 64). From our perspective, the shown positive effects of reduced cocaine use clearly favor abstinence-orientated treatment approaches of cocaine addiction. Furthermore, having the strong impact of social cognitive abilities as well as prosocial behavior and attitudes on the patient–therapist relationships in mind (15), future developments in the psychotherapy of cocaine addictions should consider trainings specifically of social skills and cognitions in order to improve treatment outcome.

## Ethics Statement

The study was approved by the Cantonal Ethics Committee of Zurich, Switzerland.

## Author Contributions

BQ and MV had full access to all the data in the study and take responsibility for the integrity of the data and the accuracy of the data analysis. CE and BQ contributed to the study concept and design. CE, MV, OB, KP, LH, MB, and BQ contributed to the acquisition, analysis, or interpretation of data. OB, CE, MV, and BQ contributed to the drafting of the manuscript. All authors contributed to the critical revision of the manuscript for important intellectual content. MV and BQ conducted the statistical analysis. ES and BQ obtained funding. KP, LH, MV, and ES contributed to the administrative, technical, or material support. ES and BQ were in charge of supervision.

## Funding

The study was supported by grants from the Swiss National Science Foundation (SNSF; grant Nos. PP00P1-123516/1 and PP00P1-146326/1) and the Olga Mayenfisch Foundation. The funders of the study did not influence the design and conduct of the study; the collection, management, analysis, and interpretation of the data; the preparation, review, and approval of the manuscript; and the decision to submit the manuscript for publication.

## Conflict of Interest Statement

The authors declare that the research was conducted in the absence of any commercial or financial relationships that could be construed as a potential conflict of interest.

## References

[1] Jovanovski D Erb S Zakzanis KK Neurocognitive deficits in cocaine users: a quantitative review of the evidence. J Clin Exp Neuropsychol (2005) 27(2):189–204. 10.1080/13803390490515694 15903150

[2] Spronk DB van Wel JH Ramaekers JG Verkes RJ Characterizing the cognitive effects of cocaine: a comprehensive review. Neurosci Biobehav Rev (2013) 37(8):1838–59. 10.1016/j.neubiorev.2013.07.003 23876288

[3] Vonmoos M Hulka LM Preller KH Jenni D Baumgartner MR Stohler R Cognitive dysfunctions in recreational and dependent cocaine users: role of attention-deficit hyperactivity disorder, craving and early age at onset. Br J Psychiatry (2013) 203(1):35–43. 10.1192/bjp.bp.112.118091 23703315

[4] Aharonovich E Nunes E Hasin D Cognitive impairment, retention and abstinence among cocaine abusers in cognitive-behavioral treatment. Drug Alcohol Depend (2003) 71(2):207–11. 10.1016/S0376-8716(03)00092-9 PMC5804498 12927659

[5] Aharonovich E Hasin DS Brooks AC Liu X Bisaga A Nunes EV Cognitive deficits predict low treatment retention in cocaine dependent patients. Drug Alcohol Depend (2006) 81(3):313–22. 10.1016/j.drugalcdep.2005.08.003 16171953

[6] Potvin S Stavro K Rizkallah E Pelletier J Cocaine and cognition: a systematic quantitative review. J Addict Med (2014) 8(5):368–76. 10.1097/ADM.0000000000000066 25187977

[7] Vonmoos M Hulka LM Preller KH Minder F Baumgartner MR Quednow BB Cognitive impairment in cocaine users is drug-induced but partially reversible: evidence from a longitudinal study. Neuropsychopharmacology (2014) 39(9):2200–10. 10.1038/npp.2014.71 PMC4104339 24651468

[8] Vonmoos M Quednow BB Cognitive dysfunctions in chronic cocaine users. In: Preedy VR The neuroscience of cocaine. San Diego: Academic Press (2017). p. 395–405. 10.1016/B978-0-12-803750-8.00040-3

[9] Rilling JK Sanfey AG The neuroscience of social decision-making. Annu Rev Psychol (2011) 62:23–48. 10.1146/annurev.psych.121208.131647 20822437

[10] Lieberman MD Social cognitive neuroscience: a review of core processes. Annu Rev Psychol (2007) 58:259–89. 10.1146/annurev.psych.58.110405.085654 17002553

[11] Couture SM Penn DL Roberts DL The functional significance of social cognition in schizophrenia: a review. Schizophr Bull (2006) 32 Suppl 1:S44–63. 10.1093/schbul/sbl029 PMC2632537 16916889

[12] Homer BD Solomon TM Moeller RW Mascia A DeRaleau L Halkitis PN Methamphetamine abuse and impairment of social functioning: a review of the underlying neurophysiological causes and behavioral implications. Psychol Bull (2008) 134(2):301–10. 10.1037/0033-2909.134.2.301 18298273

[13] Volkow ND Baler RD Goldstein RZ Addiction: pulling at the neural threads of social behaviors. Neuron (2011) 69(4):599–602. 10.1016/j.neuron.2011.01.027 21338873 PMC3188411

[14] Quednow BB Hulka LM Preller KH Baumgartner MR Eisenegger C Vonmoos M Stable self-serving personality traits in recreational and dependent cocaine users. PLoS One (2017) 12(3):e0172853. 10.1371/journal.pone.0172853 28253291 PMC5333846

[15] Quednow BB Social cognition and interaction in stimulant use disorders. Curr Opin Behav Sci (2017) 13:55–62. 10.1016/j.cobeha.2016.10.001

[16] Preller KH Hulka LM Vonmoos M Jenni D Baumgartner MR Seifritz E Impaired emotional empathy and related social network deficits in cocaine users. Addict Biol (2014) 19(3):452–66. 10.1111/adb.12070 23800218

[17] Hulka LM Eisenegger C Preller KH Vonmoos M Jenni D Bendrick K Altered social and non-social decision-making in recreational and dependent cocaine users. Psychol Med (2014) 44(5):1015–28. 10.1017/S0033291713001839 23870112

[18] Hulka LM Preller KH Vonmoos M Broicher SD Quednow BB Cocaine users manifest impaired prosodic and cross-modal emotion processing. Front Psychiatry (2013) 4:98. 10.3389/fpsyt.2013.00098 24046750 PMC3763688

[19] Preller KH Herdener M Schilbach L Stampfli P Hulka LM Vonmoos M Functional changes of the reward system underlie blunted response to social gaze in cocaine users. Proc Natl Acad Sci U S A (2014) 111(7):2842–7. 10.1073/pnas.1317090111 PMC3932870 24449854

[20] Tobler PN Preller KH Campbell-Meiklejohn DK Kirschner M Kraehenmann R Stampfli P Shared neural basis of social and non-social reward deficits in chronic cocaine users. Soc Cogn Affect Neurosci (2016) 11(6):1017–25. 10.1093/scan/nsw030 PMC4884323 26969866

[21] Frith CD Frith U Social cognition in humans. Curr Biol (2007) 17(16):R724–32. 10.1016/j.cub.2007.05.068 17714666

[22] Roepke S Vater A Preißler S Heekeren HR Dziobek I Social cognition in borderline personality disorder. Front Neurosci (2012) 6:195. 10.3389/fnins.2012.00195 23335877 PMC3543980

[23] Ruiz-Tagle A Costanzo E De Achával D Guinjoan S Social cognition in a clinical sample of personality disorder patients. Front Psychiatry (2015) 6:75. 10.3389/fpsyt.2015.00075 26074824 PMC4443650

[24] Chen KW Banducci AN Guller L Macatee RJ Lavelle A Daughters SB An examination of psychiatric comorbidities as a function of gender and substance type within an inpatient substance use treatment program. Drug Alcohol Depend (2011) 118(2–3):92–9. 10.1016/j.drugalcdep.2011.03.003 PMC3188332 21514751

[25] Fernandez-Montalvo J Lorea I Comorbilidad de la adicción a la cocaína con los trastornos de la personalidad. An Sist Sanit Navar (2007) 30(2):225–31. 10.4321/S1137-66272007000300007 17898818

[26] Albein-Urios N Martinez-Gonzalez JM Lozano-Rojas O Verdejo-Garcia A Executive functions in cocaine-dependent patients with Cluster B and Cluster C personality disorders. Neuropsychology (2014) 28(1):84–90. 10.1037/neu0000007 24219612

[27] Ford JD Gelernter J DeVoe JS Zhang W Weiss RD Brady K Association of psychiatric and substance use disorder comorbidity with cocaine dependence severity and treatment utilization in cocaine-dependent individuals. Drug Alcohol Depend (2009) 99(1–3):193–203. 10.1016/j.drugalcdep.2008.07.004 18775607 PMC2745327

[28] Lopez-Quintero C Hasin DS de Los Cobos JP Pines A Wang S Grant BF Probability and predictors of remission from life-time nicotine, alcohol, cannabis or cocaine dependence: results from the National Epidemiologic Survey on Alcohol and Related Conditions. Addiction (2011) 106(3):657–69. 10.1111/j.1360-0443.2010.03194.x PMC3227547 21077975

[29] Bagby RM Vachon DD Bulmash E Quilty LC Personality disorders and pathological gambling: a review and re-examination of prevalence rates. J Pers Disord (2008) 22(2):191–207. 10.1521/pedi.2008.22.2.191 18419238

[30] Hulka LM Vonmoos M Preller KH Baumgartner MR Seifritz E Gamma A Changes in cocaine consumption are associated with fluctuations in self-reported impulsivity and gambling decision-making. Psychol Med (2015) 45(14):3097–110. 10.1017/S0033291715001063 26081043

[31] Hoelzle C Scheufler F Uhl M Sachs H Thieme D Application of discriminant analysis to differentiate between incorporation of cocaine and its congeners into hair and contamination. Forensic Sci Int (2008) 176(1):13–8. 10.1016/j.forsciint.2007.07.020 18063333

[32] Quednow BB Kuhn KU Hoenig K Maier W Wagner M Prepulse inhibition and habituation of acoustic startle response in male MDMA (‘ecstasy’) users, cannabis users, and healthy controls. Neuropsychopharmacology (2004) 29(5):982–90. 10.1038/sj.npp.1300396 14970829

[33] Roesler M Retz W Retz-Junginger P Thome J Supprian T Nissen T Instrumente zur Diagnostik der Aufmerksamkeitsdefizit-/Hyperaktivitätsstörung (ADHS) im Erwachsenenalter. Nervenarzt (2004) 75:888–95. 10.1007/s00115-003-1622-2 15378249

[34] American Psychiatric Association (APA) Diagnostic and statistical manual of mental disorders: DSM-IV. In: Washington D, editor. American Psychiatric Association (APA)., 4th ed Washington, DC: American Psychiatric Association (APA) (1994).

[35] Dziobek I Rogers K Fleck S Bahnemann M Heekeren HR Wolf OT Dissociation of cognitive and emotional empathy in adults with Asperger syndrome using the Multifaceted Empathy Test (MET). J Autism Dev Disord (2008) 38(3):464–73. 10.1007/s10803-007-0486-x 17990089

[36] Dziobek I Fleck S Kalbe E Rogers K Hassenstab J Brand M Introducing MASC: a movie for the assessment of social cognition. J Autism Dev Disord (2006) 36(5):623–36. 10.1007/s10803-006-0107-0 16755332

[37] Charness G Rabin M Understanding social preferences with simple tests. Q J Econ (2002) 117(3):817–69. 10.1162/003355302760193904

[38] Engelmann D Strobel M Inequality aversion, efficiency, and maximin preferences in simple distribution experiments. Am Econ Rev (2004) 94(4):857–69. 10.1257/0002828042002741

[39] Linden M Lischka A-M Popien C Golombek J Der multidimensionale Sozialkontakt Kreis (MuSK)—ein Interviewverfahren zur Erfassung des sozialen Netzes in der klinischen Praxis. Z Med Psychol (2007) 16(3):135–43.

[40] Fydrich T Renneberg B Schmitz B Wittchen H-U SKID-II Strukturiertes Klinisches Interview für DSM-IV, Achse II: Persönlichkeitsstörungen. In: [SCID-II Structured Clinical Interview for DSM-IV, Axis II: Personality Disorders]. Goettingen: Hogrefe (1997).

[41] Faul F Erdfelder E Lang AG Buchner A G*Power 3: a flexible statistical power analysis program for the social, behavioral, and biomedical sciences. Behav Res Methods (2007) 39(2):175–91. 10.3758/BF03193146 17695343

[42] Christov-Moore L Simpson EA Coude G Grigaityte K Iacoboni M Ferrari PF Empathy: gender effects in brain and behavior. Neurosci Biobehav Rev (2014) 46 Pt 4:604–27. 10.1016/j.neubiorev.2014.09.001 PMC5110041 25236781

[43] Kret ME De Gelder B A review on sex differences in processing emotional signals. Neuropsychologia (2012) 50(7):1211–21. 10.1016/j.neuropsychologia.2011.12.022 22245006

[44] Wunderli MD Vonmoos M Niedecker SM Hulka LM Preller KH Baumgartner MR Cognitive and emotional impairments in adults with attention-deficit/hyperactivity disorder and cocaine use. Drug Alcohol Depend (2016) 163:92–9. 10.1016/j.drugalcdep.2016.03.026 27085500

[45] Freeman GH Halton JH Note on an exact treatment of contingency, goodness of fit and other problems of significance. Biometrika (1951) 38(1/2):141–9. 10.2307/2332323 14848119

[46] Lehrl S Mehrfachwahl-Wortschatz-Intelligenztest. MWT-B. 4th ed Balingen: Spitta (1999).

[47] Heatherton TF Kozlowski LT Frecker RC Fagerstrom KO The Fagerstrom Test for Nicotine Dependence: a revision of the Fagerstrom Tolerance Questionnaire. Br J Addict (1991) 86(9):1119–27. 10.1111/j.1360-0443.1991.tb01879.x 1932883

[48] Hautzinger M Bailer M Worall H Keller F Beck-Depressions-Inventar (BDI). Bearbeitung der deutschen Ausgabe. Testhandbuch. In: Beck Depression Inventory. Test manual., 2nd ed Bern, Göttingen, Toronto, Seattle: Huber (1994).

[49] Sussner BD Smelson DA Rodrigues S Kline A Losonczy M Ziedonis D The validity and reliability of a brief measure of cocaine craving. Drug Alcohol Depend (2006) 83(3):233–7. 10.1016/j.drugalcdep.2005.11.022 16384655

[50] Substance Abuse and Mental Health Services Administration Mandatory guidelines for federal workplace drug testing programs. Fed Regist (2008) 73(228):71858–907.

[51] Jaspers K Allgemeine Psychopathologie. 9th ed Berlin, Heidelberg, New York: Springer-Verlag (1973).

[52] American Psychiatric Association (APA) Diagnostic and statistical manual of mental disorders. 5th ed Washington, DC: American Psychiatric Association (APA) (2013) 10.1176/appi.books.9780890425596

[53] Lenzenweger MF Stability and change in personality disorder features: the longitudinal study of personality disorders. Arch Gen Psychiatry (1999) 56(11):1009–15. 10.1001/archpsyc.56.11.1009 10565501

[54] Hopwood CJ Morey LC Donnellan MB Samuel DB Grilo CM McGlashan TH Ten-year rank-order stability of personality traits and disorders in a clinical sample. J Pers (2013) 81(3):335–44. 10.1111/j.1467-6494.2012.00801.x PMC3593979 22812532

[55] Shea MT Stout R Gunderson J Morey LC Grilo CM McGlashan T Short-term diagnostic stability of schizotypal, borderline, avoidant, and obsessive-compulsive personality disorders. Am J Psychiatry (2002) 159(12):2036–41. 10.1176/appi.ajp.159.12.2036 12450953

[56] Grilo CM Sanislow CA Gunderson JG Pagano ME Yen S Zanarini MC Two-year stability and change of schizotypal, borderline, avoidant, and obsessive-compulsive personality disorders. J Consult Clin Psychol (2004) 72(5):767–75. 10.1037/0022-006X.72.5.767 PMC3289406 15482035

[57] Nestadt G Di C Samuels JF Bienvenu OJ Reti IM Costa P The stability of DSM personality disorders over twelve to eighteen years. J Psychiatr Res (2010) 44(1):1–7. 10.1016/j.jpsychires.2009.06.009 19656527 PMC2813415

[58] Durbin CE Klein DN Ten-year stability of personality disorders among outpatients with mood disorders. J Abnorm Psychol (2006) 115(1):75–84. 10.1037/0021-843X.115.1.75 16492098

[59] Lenzenweger MF Johnson MD Willett JB Individual growth curve analysis illuminates stability and change in personality disorder features: the longitudinal study of personality disorders. Arch Gen Psychiatry (2004) 61(10):1015–24. 10.1001/archpsyc.61.10.1015 15466675

[60] de Groot MH Franken IH van der Meer CW Hendriks VM Stability and change in dimensional ratings of personality disorders in drug abuse patients during treatment. J Subst Abuse Treat (2003) 24(2):115–20. 10.1016/S0740-5472(02)00351-3 12745028

[61] Harro J Neuropsychiatric adverse effects of amphetamine and methamphetamine. Int Rev Neurobiol (2015) 120:179–204. 10.1016/bs.irn.2015.02.004 26070758

[62] Rounsaville BJ Treatment of cocaine dependence and depression. Biol Psychiatry (2004) 56(10):803–9. 10.1016/j.biopsych.2004.05.009 15556126

[63] Hirsiger S Hänggi J Germann J Vonmoos M Preller KH Engeli EJE Longitudinal changes in cocaine intake and cognition are linked to cortical thickness adaptations in cocaine users. Neuroimage Clin (2019) 21:101652. 10.1016/j.nicl.2019.101652 30639181 PMC6412021

[64] Heilig M Epstein DH Nader MA Shaham Y Time to connect: bringing social context into addiction neuroscience. Nat Rev Neurosci (2016) 17(9):592–9. 10.1038/nrn.2016.67 PMC5523661 27277868

