# Supplementary material for: Improvement of Emotional Empathy and Cluster B Personality Disorder Symptoms Associated With Decreased Cocaine Use Severity
Source: Front Psychiatry. 2019 Apr 5;10:213. doi: 10.3389/fpsyt.2019.00213 (PMC6459943; doi:10.3389/fpsyt.2019.00213)
Supplement: Supplementary file 1 [file Table_1.docx]

**Supporting Information**

**Improvement of emotional empathy and cluster B personality disorder symptoms associated with decreased cocaine use severity**

Matthias Vonmoos^1,^*, PhD; Christoph Eisenegger^2,^*^,🕆^, PhD; Oliver G. Bosch^1,3^,MD;
Katrin H. Preller^1^, PhD; Lea M. Hulka^1^, PhD; Markus Baumgartner^4^, PhD; Erich Seifritz^3,,5^, MD;
Boris B. Quednow^1,,5,^*, PhD

## Methods S1. Test descriptions.

*Multifaceted Empathy Test (MET):* The MET is a reliable PC-assisted test comprising 40 photographs of people in emotionally charged situations ([1](#_ENREF_1)). The stimuli depict everyday life situations conveying information on emotional mental states via facial expression, body language and context. To measure CE, subjects are asked to infer the mental state of the person in the photograph and choose which of four words provided along with the picture describes best what the person in the picture is feeling. Explicit emotional empathy (EEE) is assessed by ratings of empathic concern (‘How concerned are you for this person?’) on a visual analog scale within a range of 1–9 (1=not concerned to 9=very concerned) while viewing the photograph. Implicit emotional empathy (IEE) is measured analogously by arousal ratings (‘How calm/aroused does this picture make you feel?’, 1=very calm to 9=very aroused). MET and MASC are implemented in Presentation (Version 14.1, Neurobehavioral Systems, Albany, CA, USA).

*Movie for the Assessment of Social Cognition (MASC):* The MASC was developed as ecologically valid and video-based multi-modal (visual and auditory input) test of social cognition ([2](#_ENREF_2)). Participants are asked to watch a 15-minute movie and make inferences about the video characters’ mental states requiring the understanding of emotions, thoughts and intentions, and concepts such as false belief, faux pas, metaphor and sarcasm in an everyday-life situation (a dinner with friends). It is paused at 45 times when questions about the actors’ feelings, thoughts and intentions are asked (‘How is Michael feeling?’). These questions are asked in a multiple-choice format with one correct answer and three distractors reflecting three different types of mistakes: (1) insufficient mental state inferences (undermentalizing: reduced ToM), (2) excessive (overmentalizing) and (3) non-mental state inferences (physical causation, no-ToM). Therefore, the MASC provides a sum score for the errors and three subscales for different error types. To control for non-social inference, memory and general comprehension effects, six control questions referring to physical events instead of a character’s mental state are asked during the test.

*Distribution/Dictator Game*: In the social interaction tasks based on ([3](#_ENREF_3), [4](#_ENREF_4)), participants were told that they would interact with two other study participants in a randomly assigned role of either player A or B. In order to warrant anonymity of the drug users, which was necessary for ethical reasons and for professional secrecy, a cover story was used where players were told that they would interact with the other subjects via Internet connection. For the purpose of this study, we were solely interested in the role of player A, wherefore player B was simulated by the computer and always responded in the same manner. The instructions specify that both players are informed about the other player’s possibilities of action and that the points will be converted into real money (Swiss Francs, CHF) at the end of the study. The plausibility of the cover story was controlled by the following question at the end of the test battery: “For reasons of anonymity you did not meet your interaction partner personally. Did you have any doubts that you interacted with someone?” Subjects responded on a five-point scale ranging from 1=*not at all* to 5=*very much*.

*Social Network Questionnaire (SNQ):* The SNQ is based on the social contact circle interview

and was designed to evaluate the size of an individual’s social network as well as the experienced emotional support and strain by this network ([5](#_ENREF_5)). Participants are required to write down the names of personal contacts in the areas ‘household’, ‘family’, ‘work or apprenticeship’, ‘friends’, ‘neighbors’ and ‘others’. Only individuals with whom they were in direct contact (via personal encounters, phone calls, emails or letters) during the previous 4 weeks should be included. Double entries of contacts are not allowed enabling the calcula-tion of the total social network size. Furthermore, participants are requested to indicate how strongly they feel emotionally supported (e.g. solace and encouragement) by their social contacts on a 5-point scale (1=not at all; 5=very much). Analogously, they specify the emotional strain (e.g. feeling of being rejected) they experience by their social contacts.

## Table S1. Socioeconomic status (number of subjects and percent).

|  | Controls (n=48) | Cocaine  Increaser (n=19) | Cocaine  Decreaser (n=19) |
| --- | --- | --- | --- |
|  |  |  |  |
| 0 - 15'000 CHF | 20 (41.7%) | 7 (36.8%) | 5 (26.3%) |
| 15'000 - 30'000 CHF | 11 (22.9%) | 3 (15.8%) | 3 (15.8%) |
| 30'000 - 60'000 CHF | 6 (12.5%) | 5 (26.3%) | 5 (26.3%) |
| 60'000 - 90'000 CHF | 5 (10.4%) | 2 (10.5%) | 6 (31.6%) |
| 90'000 - 120'000 CHF | 3 (6.3%) | 2 (10.5%) | 0 (0.0%) |
| 120'000 CHF and more | 3 (6.3%) | 0 (2.0%) | 0 (0.0%) |
|  |  |  |  |
| Fisher-Freeman-Halton Exact Test | F=10.31, p=.36 | |  |
|  |  | |  |
|  |  |  |  |

Participants were asked how much money they had available over the past year.

## Table S2. Multiple regression analyses of SCID-II Cluster B subscale change values.

|  |  |  | |  | |  |  |  |  | |  |  |  |  | |  |  |  |  | |  |  |  |  |
| --- | --- | --- | --- | --- | --- | --- | --- | --- | --- | --- | --- | --- | --- | --- | --- | --- | --- | --- | --- | --- | --- | --- | --- | --- |
|  | Histrionic  personality disorder  Δ Symptoms | | | |  | Narcisstic  personality  disorder  Δ Symptoms | | | |  | Borderline  personality  disorder  Δ Symptoms | | | |  | Antisocial  personality  disorder  Δ Symptoms | | | |  |  |  |  |  |
|  | B | | SE | β | |  | B | SE | β | |  | B | SE | β | |  | B | SE | β | |  |  |  |  |
|  |  | |  |  | |  |  |  |  | |  |  |  |  | |  |  |  |  | |  |  |  |  |
| Constant | -0.38 | | 0.84 |  | |  | 1.88 | 1.19 |  | |  | -1.98 | 1.33 |  | |  | -0.52 | 0.95 |  | |  |  |  |  |
| Age | -0.01 | | 0.02 | -0.05 | |  | -0.02 | 0.03 | -0.07 | |  | 0.01 | 0.03 | 0.03 | |  | 0.04 | 0.02 | 0.22* | |  |  |  |  |
| Sex | 0.02 | | 0.37 | 0.01 | |  | -0.91 | 0.52 | -0.18 | |  | 0.73 | 0.58 | 0.13 | |  | -0.54 | 0.42 | -0.14 | |  |  |  |  |
| ADHD-SR score | 0.00 | | 0.02 | -0.02 | |  | -0.11 | 0.04 | -0.36** | |  | -0.10 | 0.04 | -0.28* | |  | -0.02 | 0.03 | -0.07 | |  |  |  |  |
| Controls vs. Cocaine increaser | 0.03 | | 0.45 | 0.01 | |  | 0.92 | 0.64 | 0.16 | |  | 1.84 | 0.71 | 0.29* | |  | 1.21 | 0.50 | 0.28* | |  |  |  |  |
| Cocaine decreaser vs.Cocaine increaser | -0.09 | | 0.51 | -0.03 | |  | 1.03 | 0.72 | 0.18 | |  | 2.54 | 0.81 | 0.40** | |  | 0.95 | 0.57 | 0.22 | |  |  |  |  |
|  |  | |  |  | |  |  |  |  | |  |  |  |  | |  |  |  |  | |  |  |  |  |
| *R^2^* |  | | 0.00 |  | |  |  | 0.18 |  | |  |  | 0.20 |  | |  |  | 0.13 |  | |  |  |  |  |
| *F* |  | | 0.06 |  | |  |  | 3.40** |  | |  |  | 3.96** |  | |  |  | 2.20 |  | |  |  |  |  |
|  |  | |  |  | |  |  |  |  | |  |  |  |  | |  |  |  |  | |  |  |  |  |
|  |  | |  |  | |  |  |  |  | |  |  |  |  | |  |  |  |  | |  |  |  |  |

Multiple linear regression with the independent variables age, sex, ADHS-SR score, and dummy coded (zero, one) group variables.

To compare the groups, cocaine increaser acted as the reference group.

Dependent variables are change values (Δ = value at t2 - value at t1).

B = Unstandardized regression coefficient; SE = Unstandardized standard error; β = Standardized Beta. *p<.05; **p<.01; ***p<.001.

## Figure S1


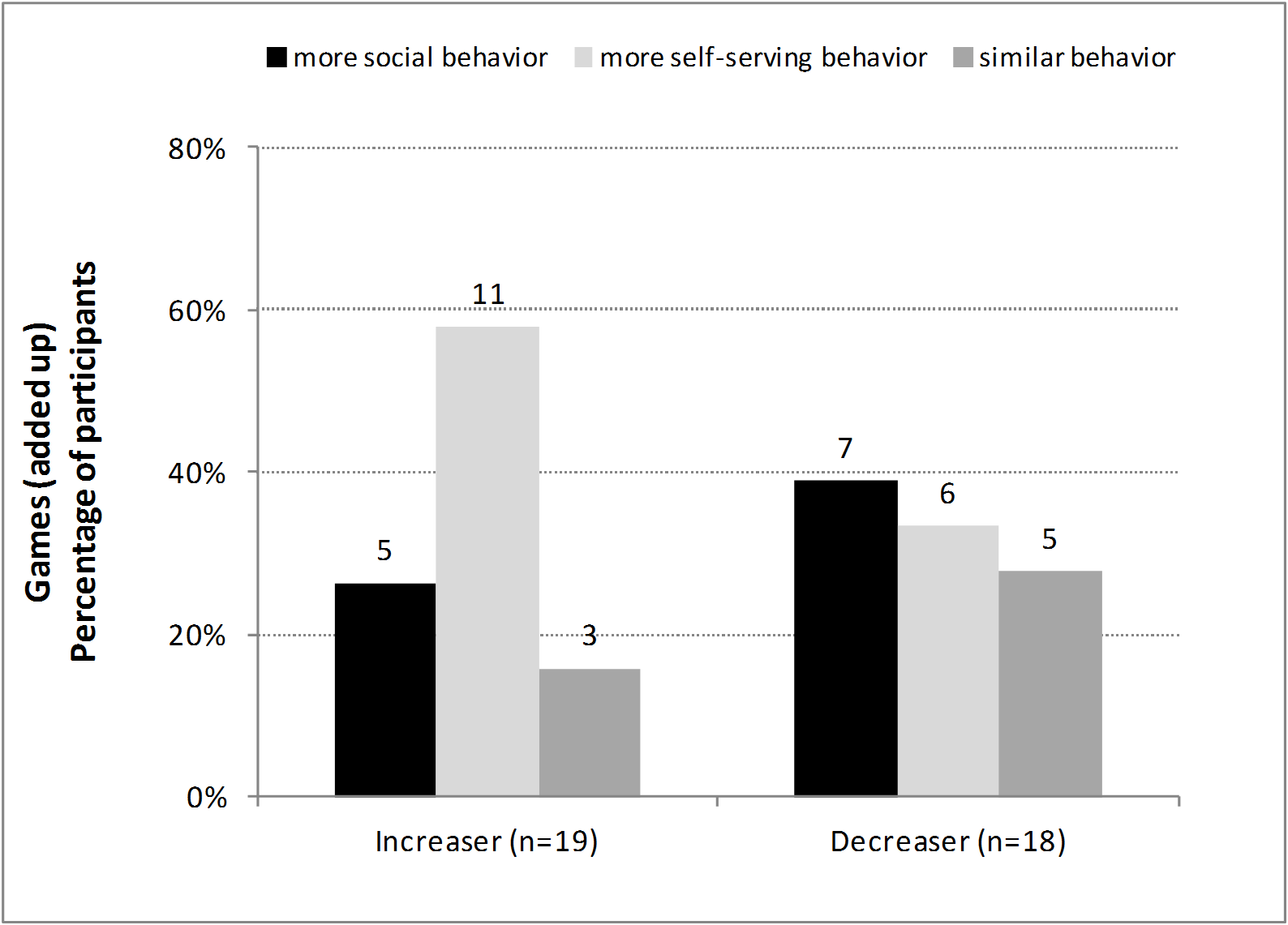


**Social behavior as measured by money distribution in cocaine *increasers*, *decreasers* and stimulant-naïve controls at baseline and follow-up.**

Distribution Game Payoff B and Dictator Game Payoff B added up. *More social behavior*: Participants gave at follow-up more money to the fictional co-player than they did at baseline. *More self-serving behavior*: Participants gave at follow-up less money to the fictional co-player B than they did at baseline. *Similar behavior*: Participants chose the same money distribution at baseline and follow-up. FET, p=.40.

## Figure S2


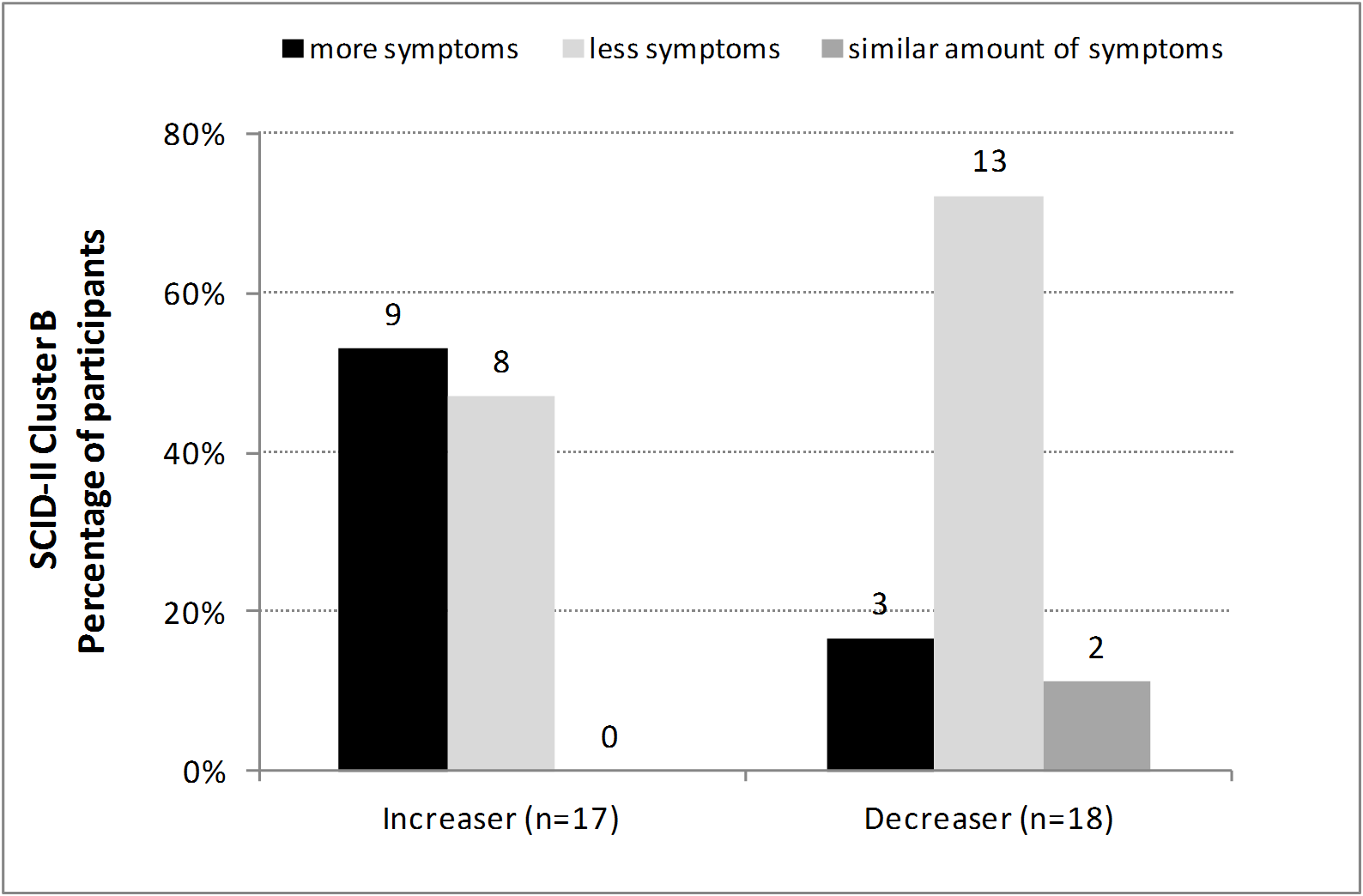


**Changes in SCID-II Cluster B symptoms in cocaine *increasers*, *decreasers* and stimulant-naïve controls within one year.**

Amount of Cluster B symptoms according to SCID-II questionnaire. *More/ less symptoms* refers to the comparison between follow-up and baseline as stated at follow-up. FET, p<.05.

## Figures S3

***a)***

***
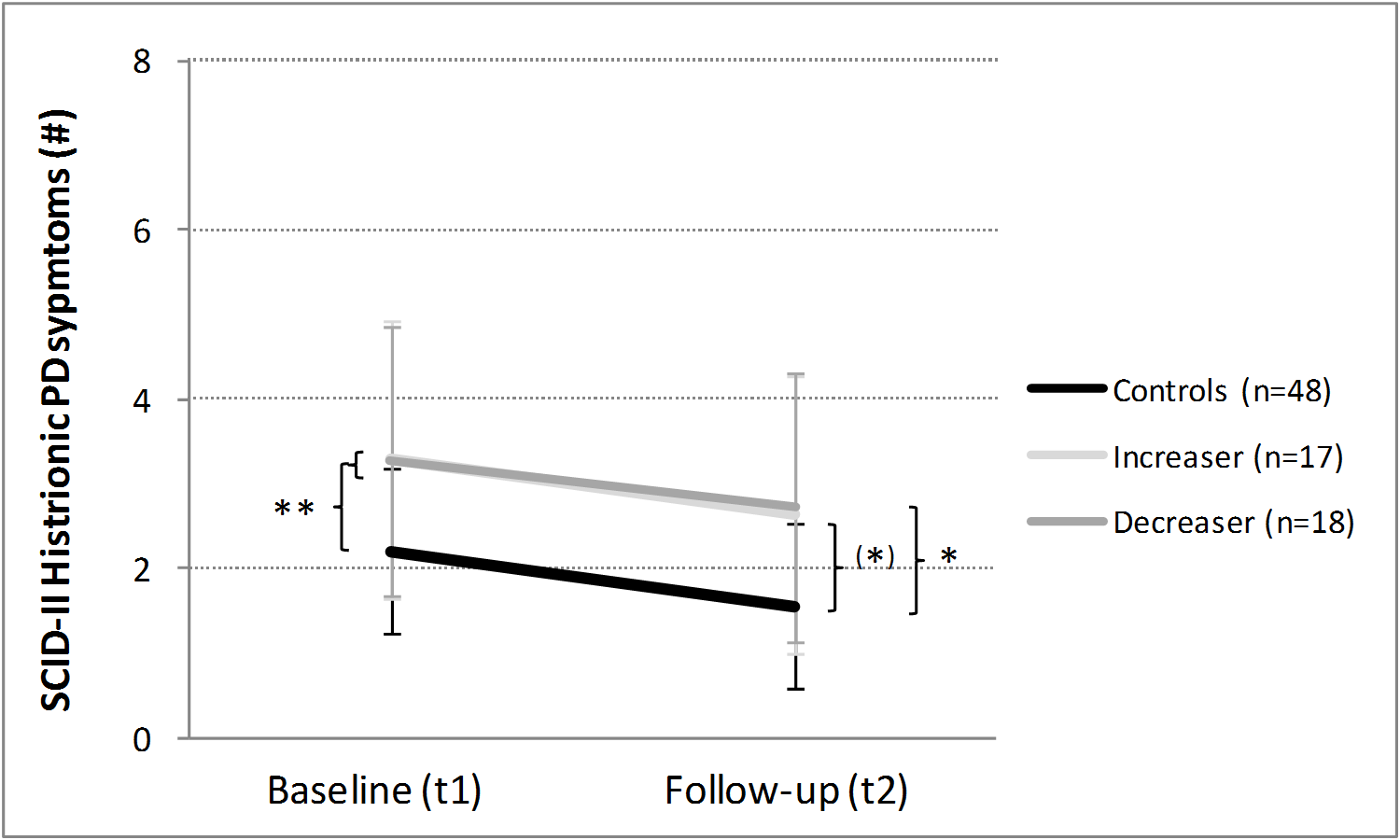
***

***b)***

***
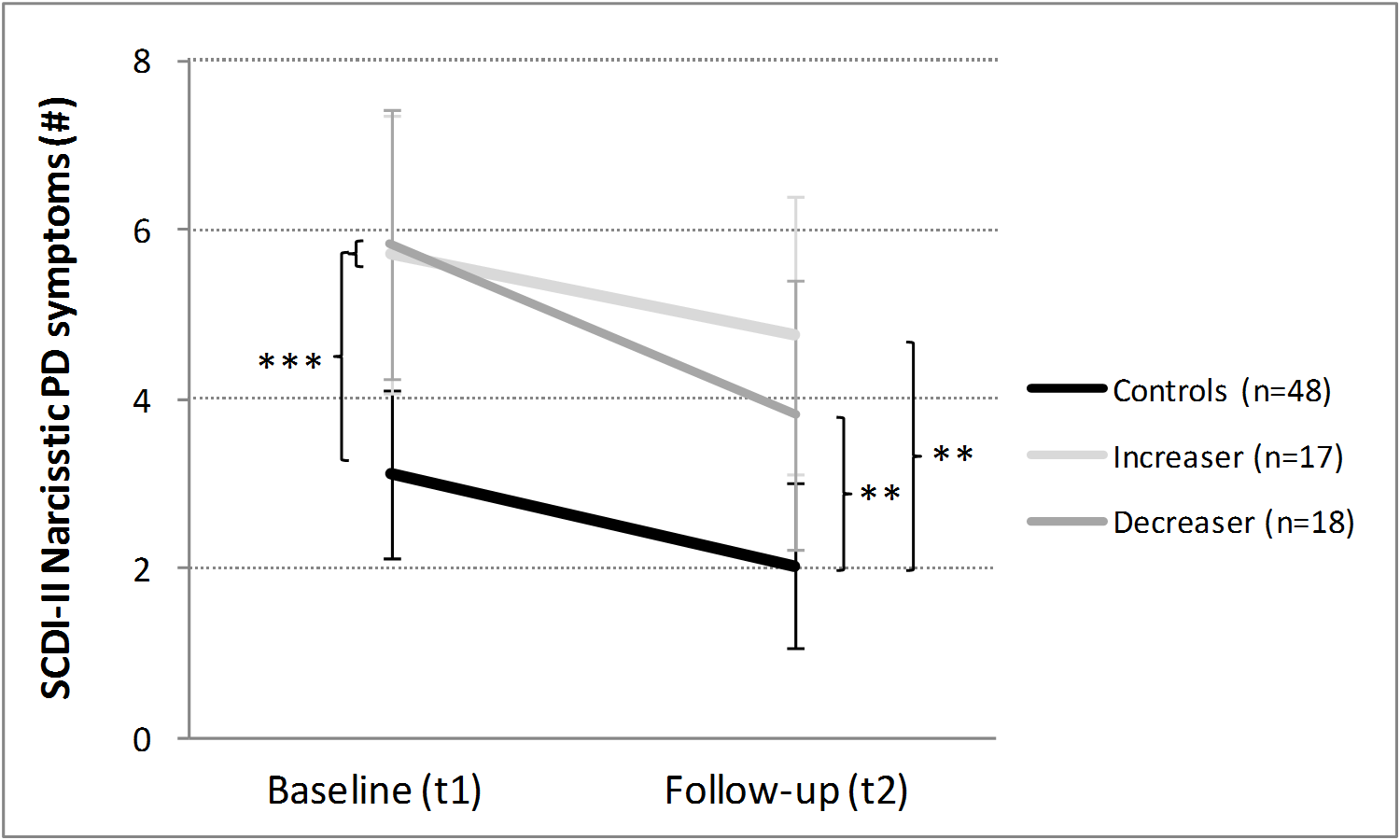
***

***c)***

***
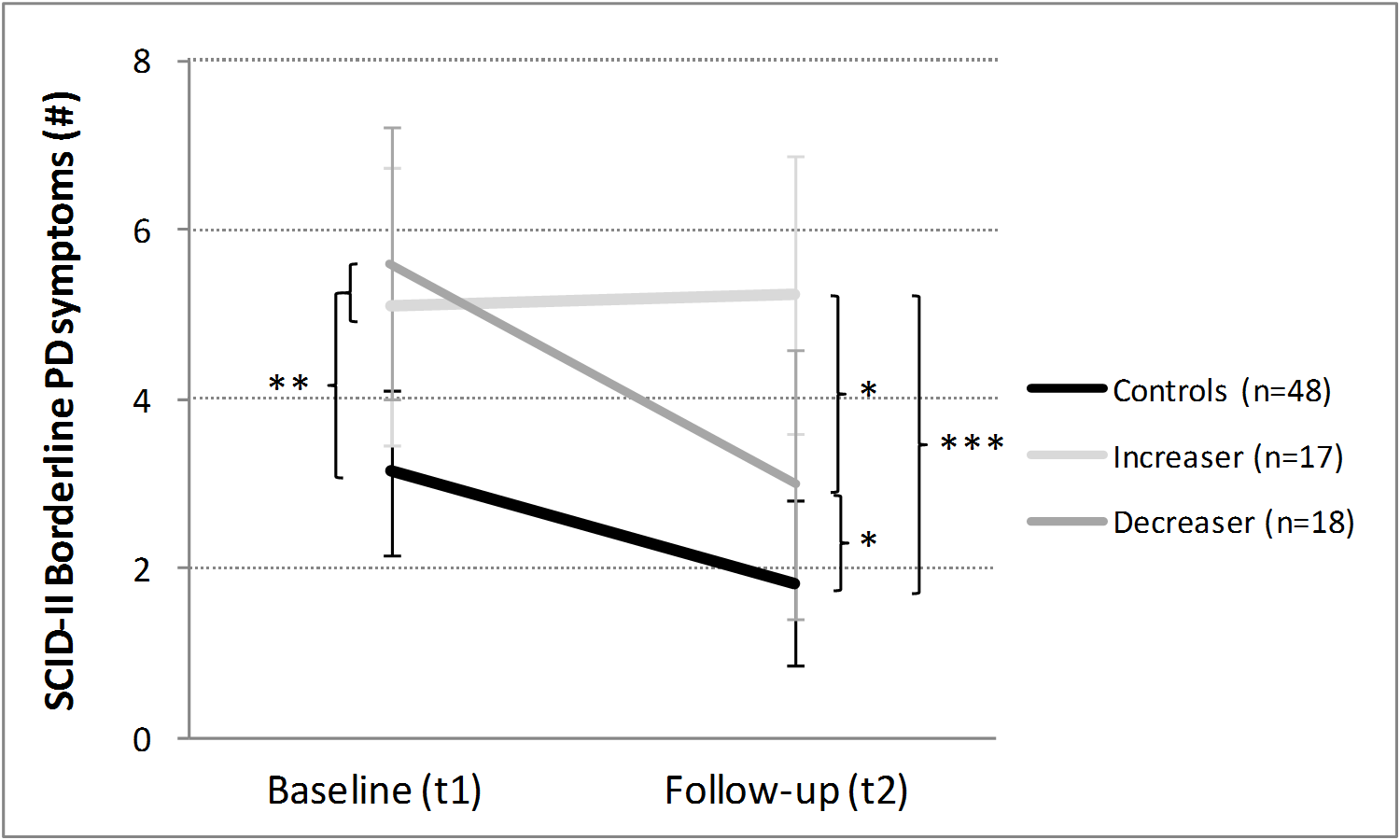
***

***d)***

***
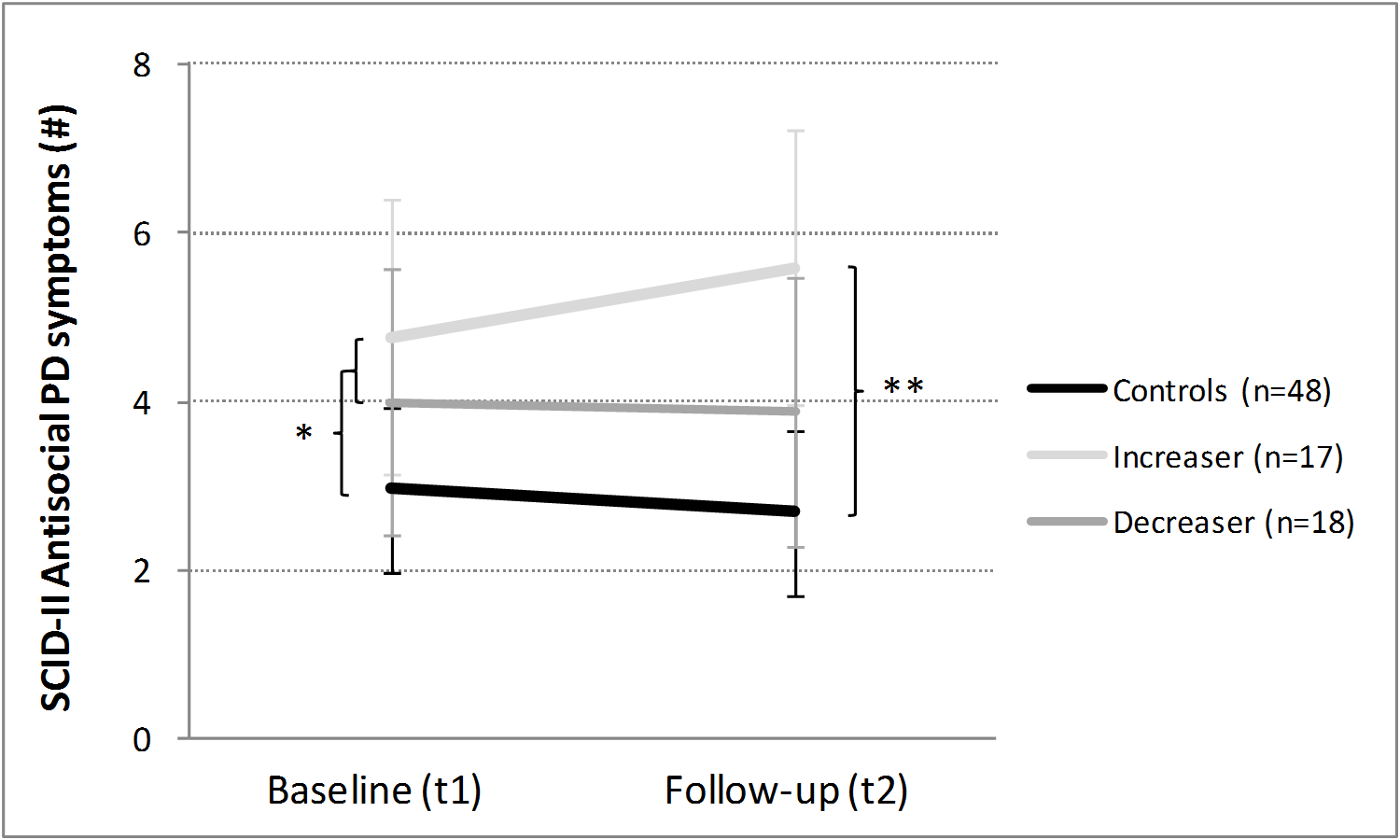
***

**Development of SCID-II a) Histrionic, b)Narcisstic, c)Borderline, d) Antisocial personality disorder in cocaine *increasers*, *decreasers* and stimulant-naïve controls within one year.**

Mean SCID-II symptoms and SE. At baseline, controls vs CCU (=Ø of increaser and decreaser). Independent Student’s t-tests are shown if p<.10. ^(^*^)^p<.10; *p<.05; **p<.01; ***p<.001.

## References

1. Dziobek I, Rogers K, Fleck S, Bahnemann M, Heekeren HR, Wolf OT, et al. Dissociation of cognitive and emotional empathy in adults with Asperger syndrome using the Multifaceted Empathy Test (MET). J Autism Dev Disord. 2008;38(3):464-73.

2. Dziobek I, Fleck S, Kalbe E, Rogers K, Hassenstab J, Brand M, et al. Introducing MASC: a movie for the assessment of social cognition. J Autism Dev Disord. 2006;36(5):623-36.

3. Charness G, Rabin M. Understanding social preferences with simple tests. Q J Econ. 2002:817-69.

4. Engelmann D, Strobel M. Inequality aversion, efficiency, and maximin preferences in simple distribution experiments. American Economic Review. 2004;94(4):857-69.

5. Linden M, Lischka A-M, Popien C, Golombek J. Der multidimensionale Sozialkontakt Kreis (MuSK) – ein Interviewverfahren zur Erfassung des sozialen Netzes in der klinischen Praxis. [The Multidimensional Social Contact Circle – an Interview for the Assessment of the Social Network in Clinicle Practical]. Z Med Psychol. 2007;16(3):135-43.
